# Effect of Cryptorchidism on the Histomorphometry, Proliferation, Apoptosis, and Autophagy in Boar Testes

**DOI:** 10.3390/ani11051379

**Published:** 2021-05-12

**Authors:** Xiaorui Fan, Yihui Liu, Meishan Yue, Weidong Yue, Gaoya Ren, Jingwen Zhang, Xinrong Zhang, Junping He

**Affiliations:** Institute of Animal Biotechnology, College of Veterinary Medicine, Shanxi Agricultural University, Taigu, Jinzhong 030801, China; m18404981554@163.com (X.F.); 18235400890@163.com (Y.L.); 18404984883@163.com (M.Y.); ywd1114@126.com (W.Y.); rgy0029618@163.com (G.R.); 18237305329@163.com (J.Z.); 18735427310@163.com (X.Z.)

**Keywords:** boar cryptorchidism, testes, histomorphometrical, proliferation, apoptosis, autophagy

## Abstract

**Simple Summary:**

Body temperature has detrimental effects on sperm quality in mammalian species, including pigs. However, the molecular mechanism of this is not yet well understood. Cryptorchidism is when one or both testes fail to descend into the scrotum, which leads the testes to be exposed to the body temperature. The aims of present study were to investigate the effect of body temperature on the histomorphometry, apoptosis and the expression of the proliferation-associated protein PCNA and the autophagy-associated protein LC3 in spontaneous unilateral cryptorchid boar testes. Our findings showed that cryptorchidism had no evident influence on the number of Sertoli cells in boars. In cryptorchid testes, spermatogonia markedly decreased and the seminiferous tubule contained only a few spermatogonia, but did not contain post-meiotic germ cells. The altered seminiferous epithelium of the cryptorchid testis showed a low proliferation of its spermatogonia, with apoptosis and autophagy like that of scrotal testis, which probably entailed a gradual degeneration of the epithelium and the impossibility of its recovery. Although the number of Sertoli cells did not change, it was likely that their functionality was altered and that this affected the proliferation capacity of spermatogonia, causing the arrest of spermatogenesis.

**Abstract:**

Spontaneous unilateral cryptorchid boars have one testis in the abdomen or inguinal canal, causing its temperature to be at or near the body temperature, which impairs spermatogenesis, although the histomorphometry and molecular mechanisms underlying this process remain unclear. The aim of the present study was to determine the histomorphometry, proliferation, apoptosis, and autophagy alterations in spermatogonia and Sertoli cells in unilateral cryptorchid, scrotal (contrascrotal), and preweaning piglet (preweaning) testes. Histomorphometrical analysis of cryptorchid testes showed that the seminiferous tubules contained only Sertoli cells and a few spermatogonia, but did not contain post-meiotic germ cells. The number of spermatogonia markedly decreased, and the number of Sertoli cells did not change remarkably in cryptorchid testes. TUNEL assay results showed that apoptosis signals were predominantly observed in spermatogonia. In cryptorchid and contrascrotal testes, proliferating cell nuclear antigen (PCNA) and LC3 were located in spermatogonia. The number of PCNA-positive, TUNEL-positive, and LC3-positive germ cells was low, and the protein and mRNA levels of PCNA and LC3 were significantly decreased in cryptorchid testes. Taken together, the number of Sertoli cells did not change remarkably, whereas the number of germ cells decreased in the cryptorchid testes, compared with that in the contrascrotal testes. Insufficient proliferation, excessive apoptosis, and autophagy were involved in the regulation of the decrease in spermatogonia in cryptorchid boar testes.

## 1. Introduction

The establishment and maintenance of germ cells are critical for ensuring the survival of a species [1]. Many factors affect germ cell maintenance, with temperature being a major factor [2,3]. Male germ cells (sperm) are produced through the complex process of spermatogenesis in the scrotum, where the temperature is lower than the core body temperature of most male mammals [4]. Testicular exposure to the body temperature leads to the arrest of spermatogenesis [5], but the detailed molecular mechanism for this remains unclear. Spontaneous unilateral cryptorchidism is a good animal model for studying the effect of body temperature on spermatogenesis.

Cryptorchidism is a congenital abnormality in which one or both testes fail to descend into the scrotal sac [6]. Its incidence in male boars is approximately 2% [7]. In cryptorchid testes, the seminiferous tubules were found to be markedly degenerated and contained spermatogonia and Sertoli cells almost exclusively [8]. In spontaneous and surgically-induced unilateral cryptorchidism in humans [9,10], rats [11], monkeys [12], rabbits [8], and rams [13], the number of germ cells was markedly decreased, whereas the number of Sertoli cells remained constant, with a slight morphological change [14]. In the spermatogenic epithelium, a tissue which contains cells that are continuously dividing, the balance of apoptosis and proliferation of testicular cells is the primary means for regulating sperm numbers and for eliminating aberrant germ cells [15]. In vivo detection of proliferating cell nuclear antigen (PCNA) or Ki-67 (a marker for mitosis) showed that the proliferative activity of germ cells decreased in unilateral cryptorchid rabbits [8], dogs [16], and boars [17]. Mice with unilateral cryptorchidism exhibited reduced testicular weight and germ cell apoptosis [18], and the blockade of caspase-2 activation prevented heat-induced testicular germ cell apoptosis in rats [19]. As an alternative death pathway to apoptosis [20], autophagy is involved in the regulation of the degeneration of the seminiferous epithelium in cryptorchidism in rats [21], mice [22], and humans [23].

We previously found that the spermatogenic epithelium of unilateral cryptorchid boar testes was severely degenerated and comprised only Sertoli cells and several spermatogonia [24]. However, no previous studies have described the histomorphometrical alterations of the germ and Sertoli cells in cryptorchid pigs, or determined whether these cell number changes are due to changes in proliferation, apoptosis, or autophagy. The present study aimed to investigate the histomorphometrical alterations in the spermatogonia and Sertoli cells of cryptorchid, contrascrotal, and preweaning piglet testes. The associations between the changes in histomorphometry and testicular cell proliferation, apoptosis, and autophagy were also investigated.

## 2. Materials and Methods

### 2.1. Animals and Sample Collection

All procedures involving the collection of testicular tissue were performed by veterinarians and in accordance with the Guiding Principles for Animal Use described by the Council for International Organizations of Medical Sciences (CIOMS) and approved by the Animal Experimentation Ethics Committee of Shanxi Agricultural University, Taigu, China.

Six unilateral cryptorchid boars (Landrace, 7–9 months of age, 140–150 kg) with undescended testes, which were considered the cryptorchidism group (cryptorchid), and with descended testes, which were considered the scrotal group (contrascrotal), and six healthy preweaning piglets collected from healthy boars (Landrace, 20 days of age, 15 kg), which were considered the preweaning piglet group (preweaning), were obtained from local farms (Taigu, Shanxi Province, China). Boars were castrated and the testes were harvested. The testicles were weighed and then the testicular tissue samples were divided into two groups. One part was fixed in Bouin’s solution for 24 h, followed by paraffin embedding. These samples were used for in situ terminal deoxynucleotidyl transferase-mediated dUTP nick-end labeling of free DNA ends (TUNEL), hematoxylin and eosin (HE) staining, and immunohistochemistry assays. The other parts were snap-frozen in liquid nitrogen and used to extract total RNA and protein for quantitative real-time polymerase chain reaction (qRT-PCR) and Western blot analysis, respectively.

### 2.2. Histomorphology

#### 2.2.1. Histology

Paraffin-embedded sections (6-μm thick) of the testes were deparaffinized with xylene and rehydrated using a graded ethanol series. Slides were stained with HE. Photomicrographs were taken using an Olympus DP71 bright-field light microscope (Olympus, Tokyo, Japan).

#### 2.2.2. Testis Morphometry

The diameter of the seminiferous tubules was measured at 200× magnification using an ocular micrometer calibrated with a stage micrometer. At least 30 seminiferous tubules with a round or nearly round morphology were selected randomly. The mean of the diameter of the seminiferous tubules was determined for each animal. Basic morphometric data on the testicular composition were obtained using the point counting method and light microscopy, using a 441-intersection grid over the sectioned material at 400× magnification. Twenty selected fields (8820 points) were randomly selected for each animal. The volume of each testicular component was determined as the product of the volume density and the testicular volume. For subsequent morphometric calculations, the specific gravity of the testis tissue was considered 1.0 [25]. To obtain a more precise measure of the testis volume, the testis capsule and mediastinum were excluded from the testis weight of the same animal [26]. The total length of the seminiferous tubules (m) was obtained by dividing the seminiferous tubule volume by the square radius time π [27]. We used the term “gonocytes” for preweaning testes since these only contain gonocytes and no spermatogonia, “spermatogonia” for cryptorchid and contrascrotal testes, and “germ cells” for preweaning, cryptorchid, and contrascrotal testes.

#### 2.2.3. Cell Counts and Cell Numbers

Sertoli cells and spermatogonia nuclei were counted in 25 round or nearly round seminiferous tubule cross-sections for each animal. The Sertoli cell nucleus was ovoid in shape, and its diameter was obtained as the mean of the larger and smaller diameters. The spermatogonial nucleus was round in shape, and its diameter was obtained as the mean of several measured diameters. To compensate for a possible overestimation of the number of Sertoli cells and spermatogonia under such conditions, initial cell counts were corrected using Abercrombie’s [28] correction factor, as follows:

Nc = N × e/(e + d)(1)

where Nc is the corrected number of cells in the preparation, N is the number of nuclei counted per tubular section, e is the thickness of the histological preparation, and d is the diameter of the nucleus of a given cell type.

The total cell numbers for Sertoli cells and spermatogonia per testis (Nt) was determined using the formula:

Nt = Lt × Nc/e(2)

where Lt denotes the length of the seminiferous tubules (estimated above), and e and Nc are defined as in Equation (1).

### 2.3. Assessment of Apoptosis

TUNEL reactions were initially conducted as described by the manufacturer using an in situ Cell Death Detection Kit, POD (Roche, Mannheim, Germany). Briefly, the paraffin-embedded testis tissue sections were deparaffinized, rehydrated, and incubated with 3% H_2_O_2_ at 37 °C for 15 min. Sections were permeabilized with a permeabilization solution (0.1% Triton-100, 0.1% sodium citrate, freshly prepared) at 37 °C for 30 min and washed two times with PBS. The sections were incubated with the TUNEL reaction mixture (50 μL of an enzyme solution in 450 μL of label solution) at 37 °C for 1 h and washed two times with PBS. Then, the sections were treated with Converter-POD (anti-fluorescein antibody, Fab fragment from sheep, conjugated with horseradish peroxidase) at 37 °C for 30 min, and washed three times with PBS. The slides were stained with 3,3′-diaminobenzidine (DAB, CWBIO) and then counterstained with hematoxylin, dehydrated, cleaned, and examined microscopically. Negative controls were prepared in the same volume of the label solution, instead of in the TUNEL reaction mixture. Only the cells with a nucleus that was stained brown were considered TUNEL-positive. At least 200 seminiferous tubules were evaluated in the cross sections of each testis, and the number of TUNEL-positive cells was determined.

### 2.4. Immunohistochemistry

Testis tissue sections were deparaffinized and rehydrated. Endogenous peroxidase activity was blocked with 3% H_2_O_2_ at 37 °C for 15 min. After washing with PBS, the sections were incubated with blocking buffer (5% BSA) at 37 °C for 30 min. The primary antibodies and incubation conditions used were as follows: polyclonal rabbit anti-PCNA antibody (1:300, Bioss, bs-2007R) and polyclonal rabbit anti-LC3 antibody (1:100, Bioss, bs-8878R) overnight at 4 °C. Sections were washed in PBS and incubated with the secondary antibody (HRP-conjugated goat-anti-rabbit; 1:100 in PBS, CWBIO, Beijing, China). The sections were washed with PBS and incubated with DAB. Finally, the sections were counterstained with hematoxylin, dehydrated, cleaned, and examined microscopically. Non-immune rabbit serum was used to determine non-specific staining. At least 200 seminiferous tubules were evaluated in the cross sections of each testis. The number of immunoreactive (nucleus/cytoplasm-stained brown) cells was determined.

### 2.5. Western Blot Analysis

Protein (20 μg) from the preweaning, cryptorchid, and contrascrotal boar testes were separated using SDS-PAGE and transferred onto nitrocellulose membranes. Membranes were blocked with blocking buffer (5% non-fat dried milk) for 30 min at room temperature, and then incubated overnight at 4 °C in blocking buffer containing polyclonal rabbit anti-PCNA antibody (1:300, Bioss, bs-2007R), polyclonal rabbit anti-LC3 antibody (1:200, Bioss, bs-8878R), and polyclonal rabbit anti-GAPDH antibody (1:2, 500, Abcam, ab9485), respectively. The next day, membranes were incubated in polymerized HRP-conjugated goat anti-rabbit secondary antibody (1:12,000) (CWBIO, Beijing, China) at 37 °C for 1 h and then washed three times with TBST. Specific protein bands were visualized and quantified using the ECL method and ImageJ software (National Institutes of Health, USA). Protein levels were corrected for GAPDH expression levels. All experiments were performed in triplicate.

### 2.6. Total RNA Extraction and Quantitative Real-Time PCR Analysis

Total RNA was extracted from the homogenized boar testis tissue using TRIzol reagent (Invitrogen, USA), according to the manufacturer’s protocol. The concentration and quantity of total RNA were determined using a spectrophotometer (NanoDrop ND-1000, USA). The relative expression levels of PCNA and LC3 mRNAs were validated using qRT-PCR. Thereafter, 1 μg of total RNA from the testicular tissue was reverse transcribed using the Prime ScriptTM RT reagent kit (TaKaRa, Dalian, China) according to the manufacturer’s protocol. qRT-PCR was performed with the SYBR^®^ Premix Ex TaqTM II (TaKaRa, Dalian, China) using triplicates for each sample with the Stratagene Mx3005P qPCR system (Stratagene Agilent, Santa Clara, CA, USA). The primers used for PCNA and LC3 were designed using Primer3Plus (http://www.primer3plus.com/cgi-bin/dev/primer3plus.cgi, accessed on 12 May 2021). 18S rRNA was used as an endogenous control. The primers used were as follows: PCNA (forward 5′-CCTGAAGAAGGTGCTGGAA-3′ and reverse 5′-TTCTGCCCTTAGCGTAATGAT-3′); LC3 (forward 5′-CCTCAGACCGGCCTTTCA-3′ and reverse 5′-TCATCCTTCTCCTGCTCATAG-3′); and 18S rRNA (forward 5′-CCCACGGAATCGAGAAAGAG-3′ and reverse 5′-TTGACGGAAGGGCACCA-3′). The 2^−ΔΔCt^ method was used to estimate the relative expression levels of the genes, as previously described [28]. Each graph represents at least three independent experiments.

### 2.7. Statistical Analysis

Statistical analysis was performed using SPSS software (version 16.0; SPSS Inc., Chicago, IL, USA), and the results were transformed to logarithms for one-way ANOVA. All data are presented as mean ± SD. The significance level was set at *p* < 0.05.

## 3. Results

### 3.1. Histological Changes in Boar Testes

**Figure 1:**
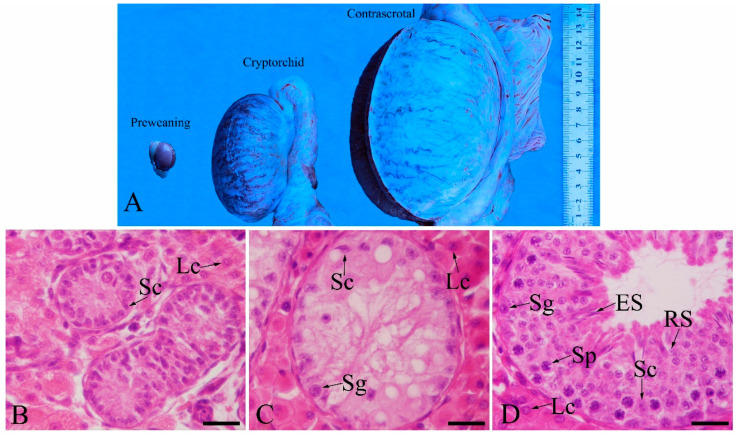
Testicular sizes and HE staining of boar testes. Testicular sizes, preweaning testes, cryptorchid testes, and contrascrotal testes (**A**–**D**). Sg, spermatogonia; Sp, spermatocyte; RS, round spermatid; ES, elongated spermatid; Sc, Sertoli cell; Lc, Leydig cell. Bar = 25 μm.

Histological analyses were performed to examine the changes in the preweaning, cryptorchid and contrascrotal testes. Cryptorchid testes were markedly smaller than the contrascrotal testes (Figure 1A). In preweaning testes, round-to-oval Sertoli cell nuclei and gonocytes were located within the cord and/or close to the basal lamina. No lumen was detected within the cord (Figure 1B). In the cryptorchid boar testis, the spermatogenic epithelium comprised only Sertoli cells and several spermatogonia but did not contain post-meiotic germ cells (Figure 1C). Contrascrotal boar testes showed normal histology, and multiple types of germ cells, including spermatogonia, spermatocytes, round spermatids, and elongated spermatids, and Sertoli cells were observed in the spermatogenic epithelium (Figure 1D).

### 3.2. Histomorphometric Changes of Boar Testes

Compared to the preweaning testes, the testicular weight, length, and diameter of the seminiferous tubules were significantly increased (*p* < 0.01) in cryptorchid testes. The weight, length, and diameter of the seminiferous tubules of the cryptorchid testis were significantly decreased (*p* < 0.01) compared with the contrascrotal testis (Table 1). The mean testicular weight values of the preweaning, cryptorchid, and contrascrotal testes in boars were 4.17, 68.41, and 311.06 g, respectively. The volume density values of the seminiferous tubules of preweaning, cryptorchid, and contrascrotal testes were approximately 38%, 66%, and 69%, respectively. The mean tubule diameter was 49.19, 122.74, and 255.35 μm in preweaning, cryptorchid, and contrascrotal testes, respectively. Based on the volume of the testis parenchyma (testis weight minus the testis capsule and mediastinum) and the volume occupied by seminiferous tubules in the testis, as well as the diameter of the seminiferous tubules, approximately 474.07, 3331.27, and 4837.65 m of the total length of seminiferous tubules were observed in the preweaning, cryptorchid, and contrascrotal testes, respectively (Table 1).

**Figure 2:**
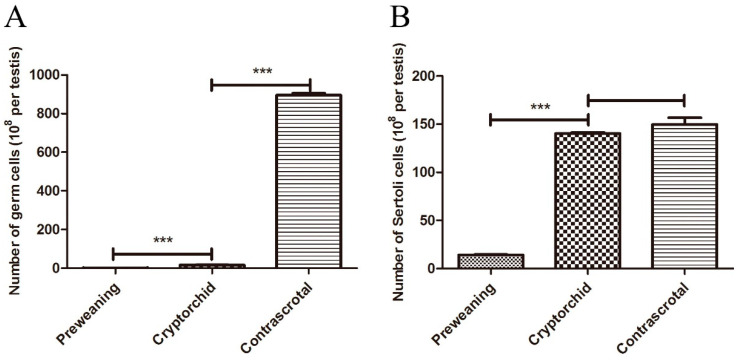
The number of germ and Sertoli cells in seminiferous tubules of boar testes. The number of germ and Sertoli cells (**A**,**B**) in preweaning, cryptorchid, and contrascrotal testes. *** *p* < 0.001.

The total number of germ cells per cryptorchid testis was significantly increased (*p* < 0.001) compared with that in the preweaning testes. However, the total number of germ cells per cryptorchid testis was decreased by approximately 55-fold (*p* < 0.001) compared with that in the contrascrotal testis, suggesting that the number of germ cells was significantly decreased in the cryptorchid testes (Figure 2A). Compared to the preweaning testis, the total number of Sertoli cells per cryptorchid testis was significantly increased (*p* < 0.001). The total number of Sertoli cells per cryptorchid testis did not change compared with that in the contrascrotal testis (Figure 2B).

### 3.3. Apoptosis, Proliferation, and Autophagy Changes in Boar Testes

#### 3.3.1. Qualitative Assessment of Apoptosis

**Figure 3:**
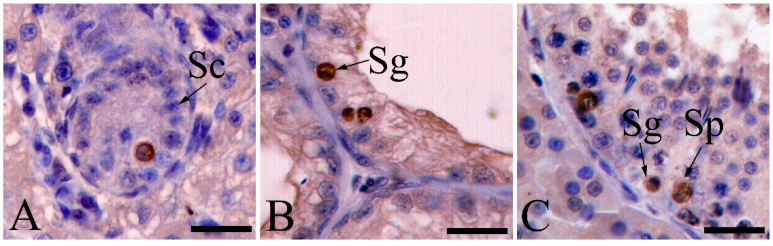
TUNEL detection of apoptotic nuclei in seminiferous tubules of boar testes. Apoptosis in preweaning, cryptorchid, and contrascrotal testes (**A**–**C**). Nuclei of apoptotic cells were stained dark brown. Sg, spermatogonia; Sp, spermatocyte; Sc, Sertoli cell. Bar = 25 μm.

**Figure 4:**
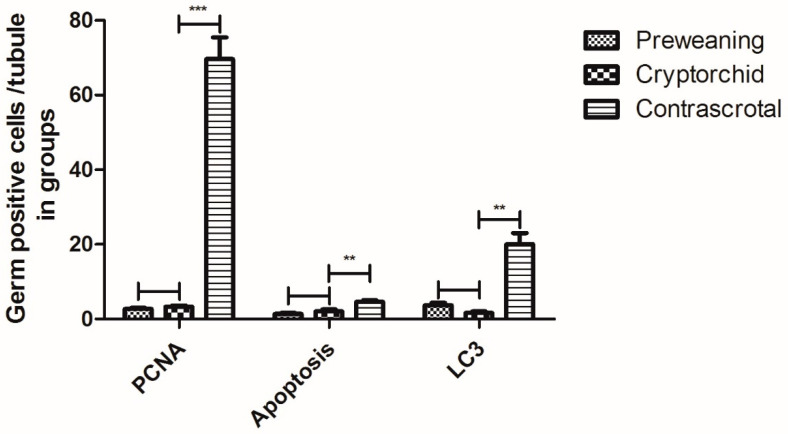
Germ-positive cells in each seminiferous tubule of preweaning, cryptorchid, and contrascrotal boar testes. ** *p* < 0.01, *** *p* < 0.001.

Apoptosis was detected using the TUNEL assay in preweaning, cryptorchid, and contrascrotal testes. In the preweaning testes, the TUNEL-positive cells included gonocytes (Figure 3A). TUNEL-positive spermatogonia were observed in cryptorchid testes (Figure 3B). In contrast, germ cell apoptosis was observed primarily in spermatogonia and several spermatocytes (Figure 3C). The number of TUNEL-positive germ cells in cryptorchid testes (1.17 ± 0.50) decreased (*p* < 0.01) compared with that in the contrascrotal testes (3.18 ± 1.30) (Table 2, Figure 4).

#### 3.3.2. PCNA and LC3 Immunohistochemical Localization in Boar Testes

**Figure 5:**
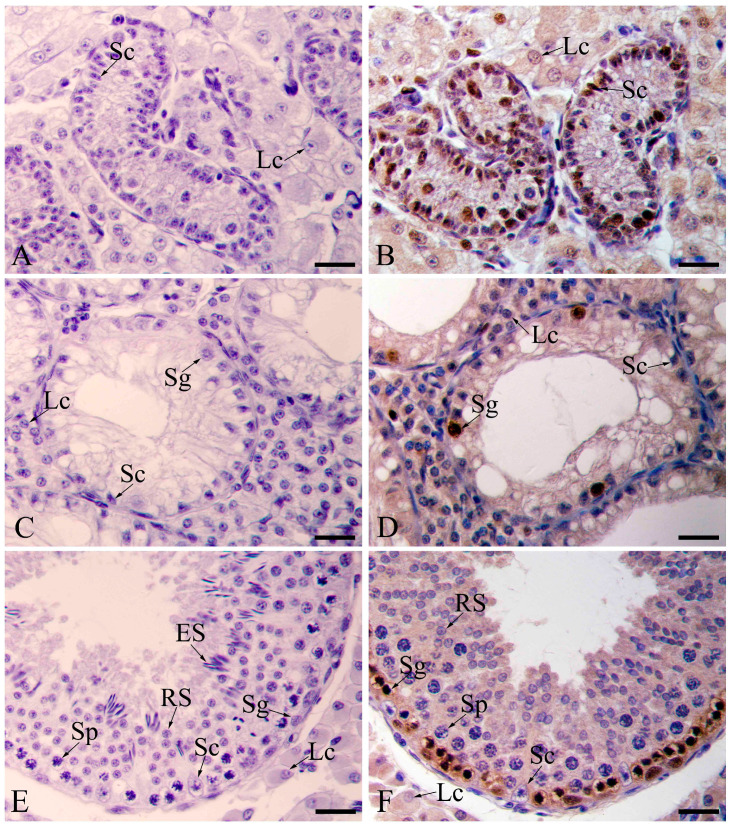
Immunohistochemical staining of PCNA in seminiferous tubules of boar testes. PCNA-positive cells in preweaning, cryptorchid, and contrascrotal testes—(**B**,**D**,**F**), respectively. In the negative control sections of boar testes—(**A**,**C**,**E**)—non-immune rabbit serum was substituted for the primary antibody in the first reaction. Note the lack of immunoreactivity. Sg, spermatogonia; Sp, spermatocyte; RS, round spermatid; ES, elongated spermatid; Sc, Sertoli cell; Lc, Leydig cell. Bar = 25 μm.

Immunohistochemical analysis and tests were performed to determine the distribution of proliferation protein PCNA. In preweaning testes, PCNA was located in the nuclei of gonocytes, Sertoli cells, and Leydig cells (Figure 5B). The immunostaining results were positive in the spermatogonia and Leydig cells in cryptorchid testes, and negative in Sertoli cells (Figure 5D). A large number of PCNA-positive spermatogonia was observed in the contrascrotal testes, and Sertoli cells did not show a positive PCNA expression (Figure 4F). Negative control sections did not show positive staining (Figure 5A,C,E).

The number of PCNA-positive germ cells in cryptorchid testes (3.29 ± 0.70) decreased (*p* < 0.001) compared to that in the contrascrotal testes (69.67 ± 3.18) (Table 2, Figure 4).

**Figure 6:**
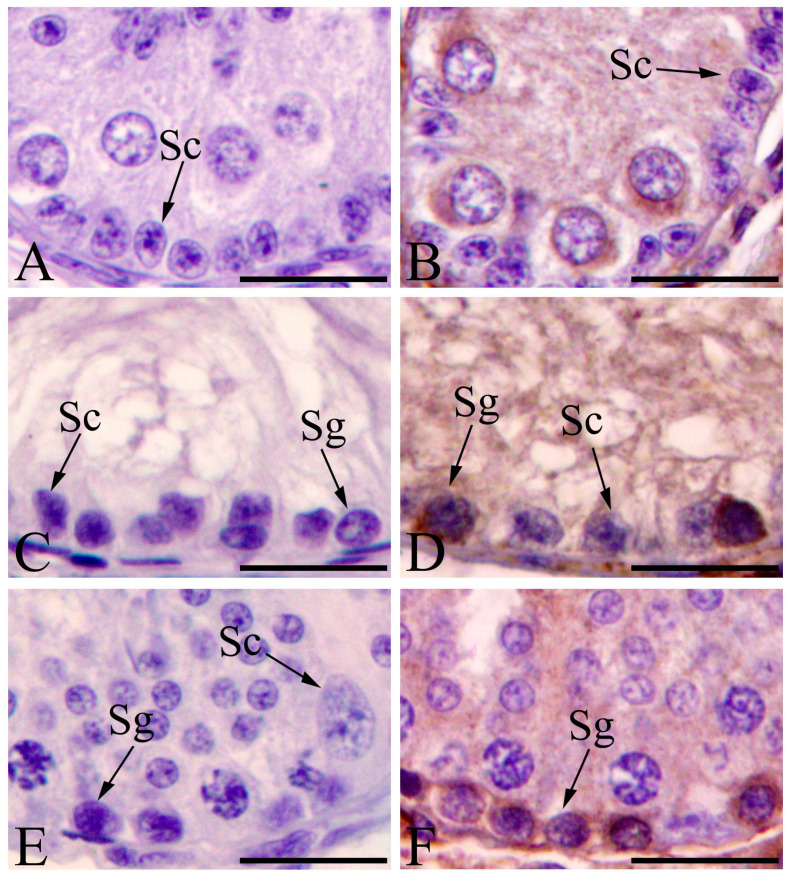
Immunohistochemical staining of LC3 in seminiferous tubules of boar testes. LC3-positive cells in preweaning, cryptorchid, and contrascrotal testes—(**B**,**D**,**F**), respectively. In the negative control sections of boar testes—(**A**,**C**,**E**)—non-immune rabbit serum was substituted for the primary antibody in the first reaction. Note the lack of immunoreactivity. Sg, spermatogonia; Sc, Sertoli cell. Bar = 25 μm.

Immunohistochemical staining was carried out to test for the distribution of the autophagy regulation protein LC3. LC3 was expressed in the cytoplasm of gonocytes in the preweaning testes (Figure 6B). LC3 was immunolocalized in the spermatogonia in the cryptorchid and contrascrotal testes (Figure 6D,F). No positive staining was observed in the negative control sections (Figure 6A,C,E).

The number of LC3-positive germ cells in the cryptorchid testes (2.15 ± 0.66) decreased (*p* < 0.01) compared with that in the contrascrotal testes (19.40 ± 0.53) (Table 2, Figure 4).

### 3.4. Western Blot Analysis of PCNA and LC3 in Boar Testes

**Figure 7:**
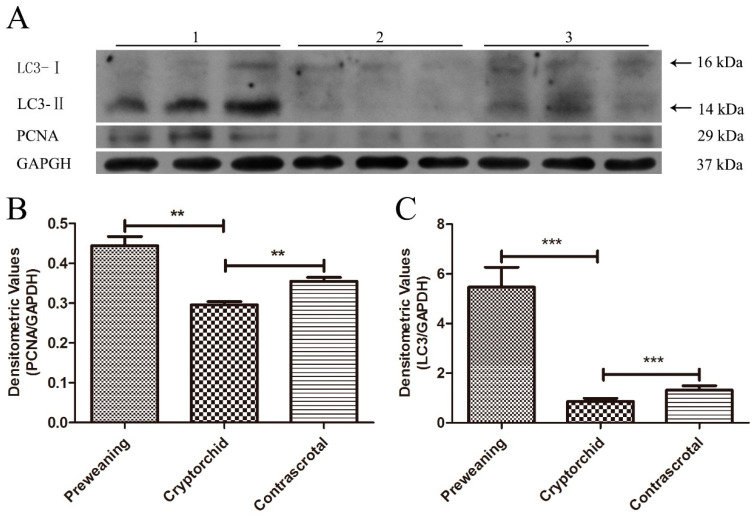
Western blot analysis for PCNA and LC3 protein in boar testes. GAPDH was used as an internal control. Western blot results of PCNA and LC3 in preweaning, cryptorchid, and contrascrotal testes (**A**). The histograms represent the densitometric values of the immunoblots, (**B**,**C**). 1, preweaning; 2, cryptorchid; 3, contrascrotal. ** *p* < 0.01, *** *p* < 0.001.

In preweaning, cryptorchid, and contrascrotal testes, the protein expression of PCNA and LC3 was detected using Western blot analysis (Figure 7A). The expression of PCNA and LC3 in the cryptorchid testes (0.30 ± 0.01 and 0.86 ± 0.10, respectively) was significantly decreased (*p* < 0.01 and *p* < 0.001, respectively) compared with that in the preweaning testes (0.44 ± 0.03 and 5.47 ± 0.65, respectively). The expression of PCNA and LC3 in the cryptorchid testes (0.30 ± 0.01 and 0.86 ± 0.10, respectively) was significantly decreased (*p* < 0.01 and *p* < 0.001, respectively) compared with that in the contrascrotal testes (0.36 ± 0.01 and 1.32 ± 0.14, respectively) (Figure 7B,C and Table 3).

### 3.5. Expression of PCNA and LC3 mRNA in Boar Testes

**Figure 8:**
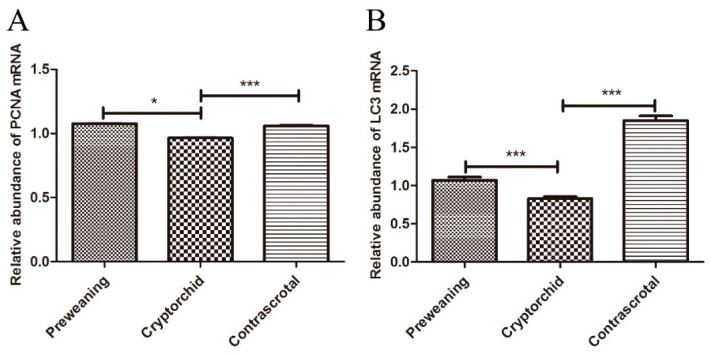
Relative expression levels of PCNA and LC3 mRNA in boar testes. Expression levels of PCNA and LC3 (**A**,**B**) mRNA in preweaning, cryptorchid, and contrascrotal testes. * *p* < 0.05, *** *p* < 0.001.

qRT-PCR was used to analyze the expression of PCNA and LC3 mRNA in preweaning, cryptorchid, and contrascrotal testes. The mRNA expression of PCNA in cryptorchid testes (0.96 ± 0.01) was significantly decreased (*p* < 0.05) compared to that in the preweaning testes (1.08 ± 0.01). Compared with that in the contrascrotal testes (1.06 ± 0.01), the level of PCNA mRNA was significantly decreased (*p* < 0.001) in the cryptorchid testes (0.96 ± 0.01). LC3 mRNA expression in the cryptorchid testes (0.83 ± 0.02) was significantly decreased (*p* < 0.001) compared to that in preweaning testes (1.07 ± 0.03). Compared with that in the contrascrotal testes (1.85 ± 0.05), the level of LC3 mRNA was significantly decreased (*p* < 0.001) in the cryptorchid testes (0.83 ± 0.02) (Figure 8A,B and Table 3).

## 4. Discussion

The present study demonstrates the histomorphometrical alterations of germ and Sertoli cells in unilateral cryptorchid, contrascrotal, and preweaning piglet testes. The associations among the changes in testicular cell proliferation, apoptosis, and the autophagy index were also investigated. In unilateral cryptorchid testes, the seminiferous tubule contained Sertoli cells and several spermatogonia but did not contain post-meiotic germ cells. Sertoli cells and multiple types of germ cells, including spermatogonia, spermatocytes, and spermatids, were found in the contrascrotal testes. Only Sertoli cells with oval nuclei and gonocytes were detected in the testicular tissue of preweaning piglets, and the lumen was not identified. Similar morphological alterations have been reported in spontaneous and surgically induced unilateral cryptorchidism rats [29], boars [15], horses [30], dogs [31], and rabbits [8], in which the spermatogenic epithelium of the cryptorchid testes comprised only Sertoli cells and several spermatogonia and did not contain post-meiotic germ cells.

The number of Sertoli cells was not significantly different between cryptorchid and contrascrotal testes, the number of germ cells markedly decreased, and there were no post-meiotic spermatocytes in cryptorchid testes. Similar results were found in spontaneous and surgically induced unilateral cryptorchidism in humans [9,10], rats [11], monkeys [12], rabbits [8], and rams [13]. The number of germ cells was markedly decreased, whereas the number of Sertoli cells remained constant, with a slight morphological change [14]. Compared with the preweaning piglet testes, in the cryptorchid testes, the number of Sertoli and germ cells increased, the gonocytes differentiated into spermatogonia, and the testis cords developed into seminiferous tubules. These results are consistent with previous findings on cryptorchidism in horses [30] and dogs [31], suggesting that cryptorchidism did not affect the formation of seminiferous tubules and the differentiation of gonocytes into spermatogonia.

In the present study, the protein and mRNA levels of PCNA were found to be decreased in cryptorchid testes compared to those in contrascrotal testes. Numerous PCNA-positive Sertoli cells were identified in preweaning piglet testes, and high cell proliferation was a characteristic feature of the immature testes. The proliferative activity of Sertoli cells was not identified in either of the lateral testes, which was in agreement with the results of a previous study on rats [32] and pigs [26]. This suggests that Sertoli cells do not proliferate after puberty in boars. The results indicate that the number of Sertoli cells was not significantly different between cryptorchid and contrascrotal testes, and there were no remarkable effects of cryptorchidism on the proliferation of Sertoli cells. PCNA-positive Leydig cells were identified in preweaning piglet testes and cryptorchid testes. Leydig cells are responsible for the production of testosterone (T). T is required for sexual development and testis descent during the fetal period, as well as the production of sperm in the seminiferous tubules at adulthood [33]. The proliferative activity of Leydig cells was identified in cryptorchid testes, which suggested that the Leydig cells secrete T as much as possible to support the development of residual germ cells in cryptorchid boar testes.

In this study, the immunohistochemical localization of PCNA was found in the spermatogonia in both cryptorchid and contrascrotal testes, and the number of PCNA-positive germ cells was decreased in cryptorchid testes. A decrease in spermatogonium proliferation activity was observed in spontaneous unilateral cryptorchid boys [34], dogs [16], and boars [15], as well as in an experimentally induced cryptorchid rabbit model [8]. Although the proliferation activity decreased, the low proliferative activity suggested that the number of spermatogonia increased via proliferation in the cryptorchid testes. However, no post-meiotic spermatocytes were observed in the cryptorchid boar testes. Combined with the results of our previous study, it was determined that spermatogonia could enter meiosis, but no post-meiotic germ cells were found in the cryptorchid boar testes, and the degeneration of post-meiotic germ cells might be due to apoptotic signals or other unexplored reasons [23].

In the present study, DNA strand breaks in apoptotic cells were detected using the TUNEL assay. There were no TUNEL-positive Sertoli cells in the preweaning, cryptorchid, or contrascrotal boar testes. The number of apoptotic positive germ cells was lower in cryptorchid testes than in contralateral testis. Similar results were found in cryptorchid rats [35] and boars [15]. Additionally, the number of apoptotic cells was considerably increased in short-term scrotal hyperthermia-treated rat testes, whereas it was decreased in long-term scrotal hyperthermia-treated ones because of the decrease in the total number of spermatogenic cells [36]. In summary, the cryptorchid testes showed a decrease in germ cell apoptosis due to the cumulative decrease in the total number of spermatogenic cells. Apoptosis is an important mechanism for the further reduction of germ cells in cryptorchid boars.

Autophagy is a compensatory process for the removal of impaired cells in organisms. LC3, which is a reliable protein marker, has been widely used to monitor autophagy [37]. In the present study, the protein and mRNA levels of LC3 decreased in cryptorchid testes compared to those in contrascrotal testes. No positive immunohistochemical expression of LC3 was detected in the Sertoli cells of preweaning, cryptorchid, or contrascrotal boar testes, and several LC3-positive spermatogenic cells were detected in preweaning, cryptorchid, and contrascrotal boar testes. Autophagy, which is involved in the removal of impaired spermatogenic cells in cryptorchid testes, has been reported in humans [21], rats [22], and mice [23]. Therefore, autophagy also serves to reduce the number of germ cells in cryptorchid boars.

## 5. Conclusions

Cryptorchism had no evident influence on the number of Sertoli cells in boars. In unilateral cryptorchid testes, the number of germ cells was markedly decreased, and the seminiferous tubules contained only a few spermatogonia but did not contain post-meiotic germ cells. The number of PCNA-positive, TUNEL-positive, and LC3-positive germ cells was low, and the protein and mRNA levels of PCNA and LC3 were significantly decreased in cryptorchid testes. Insufficient proliferation, excessive apoptosis, and autophagy were involved in the regulation of the decrease in germ cells in cryptorchid boar testes. Furthermore, although the number of Sertoli cells did not change, it was likely that their functionality was altered and affected the proliferation capacity of spermatogonia, causing the arrest of spermatogenesis.

## Figures and Tables

**Table 1:** Quantitative data regarding the main testicular structures in boars in the preweaning, cryptorchid and contrascrotal groups. The results represent mean ± SD.

Parameter	Age (20 Days)	Age (7–9 Months)	
	Preweaning (n = 6)	Cryptorchid (n = 6)	Contrascrotal (n = 6)
Testis weight (g)	4.17 ± 0.12 ^f^	68.41 ± 9.54 ^e^	311.06 ± 10.06 ^d^
Seminiferous tubule (%) ^a^	38.83 ± 1.57 ^e^	66.88 ± 8.22 ^d^	69.07 ± 4.03 ^d^
Seminiferous tubules:			
Diameter (μm)	49.19 ± 1.80 ^f^	122.74 ± 1.78 ^e^	225.35 ± 1.61 ^d^
Length (m per testis)	474.07 ± 17.80 ^f^	3331.27 ± 81.15 ^e^	4837.65 ± 276.21 ^d^
Germ cell:			
No. per cross-section ^b^	1.04 ± 0.08 ^e^	2.92 ± 0.17 ^e^	115.61 ± 1.91 ^d^
No. per Testis (×10^8^)	0.84 ± 0.03 ^e^	16.2 ± 0.94 ^e^	895.41 ± 17.13 ^d^
Sertoli cell:			
Nuclei per cross-section ^c^	17.78 ± 0.51 ^e^	25.96 ± 0.30 ^e^	19.85± 1.36 ^d^
Nuclear diameter (μm)	6.37 ± 0.12 ^e^	6.45 ± 0.49 ^e^	8.41 ± 0.47 ^d^
No. per Testis (×10^8^)	14.05 ± 0.72 ^e^	140.26 ± 1.58 ^d^	149.55 ± 10.26 ^d^

^a^ Percentage occupied by seminiferous tubule in the testicular parenchyma. ^b^ Total corrected number of germ cells per seminiferous cord/tubule cross-section. ^c^ Crude number of Sertoli cell nuclei per seminiferous cord/tubule cross-section. ^d,e,f^ Means in rows with different superscripts differ (*p* < 0.01 to *p* < 0.001).

**Table 2:** Expression of PCNA, LC3, and apoptosis in germ cells and Sertoli cells of preweaning, cryptorchid, and contrascrotal testes, respectively. The results represent mean ± SD.

Group (n = 6)	Expression of PCNA		Apoptosis		Expression of LC3	
	Germ Cells	Sertoli Cells	Germ Cells	Sertoli Cells	Germ Cells	Sertoli Cells
Preweaning	2.83 ± 0.37 ^b,1^	17.00 ± 1.15	1.05 ± 0.23 ^b^	0	3.67 ± 0.94 ^b,3^	0
Cryptorchid	3.29 ± 0.70 ^b,2^	0	1.17 ± 0.50 ^b^	0	2.15 ± 0.66 ^b^	0
Contrascrotal	69.67 ± 3.18 ^a,2^	0	3.18 ± 1.30 ^a^	0	19.40 ± 0.53 ^a,4^	0

^a,b^ Means in same column with different superscripts differ (*p* < 0.05 to *p* < 0.001). ^1^ Significant different expression of PCNA vs. apoptosis of germ cells in preweaning, cryptorchid, and contrascrotal testes (*p* < 0.05). ^2^ Significantly different expression of PCNA vs. apoptosis of germ cells in preweaning, cryptorchid, and contrascrotal testes (*p* < 0.01). ^3^ Significantly different expression of LC3 vs. apoptosis of germ cells in preweaning, cryptorchid and contrascrotal testes (*p* < 0.05). ^4^ Significantly different expression of LC3 vs. apoptosis of germ cells in preweaning, cryptorchid, and contrascrotal testes (*p* < 0.01).

**Table 3:** Expression of PCNA and LC3 proteins and mRNA in preweaning, cryptorchid, and contrascrotal, respectively. The results represent mean ± SD.

Group (n = 6)	Expression of PCNA and LC3 Protein		Expression of PCNA and LC3 mRNA	
	PCNA	LC3	PCNA	LC3
Preweaning	0.44 ± 0.03 ^c^	5.47 ± 0.65 ^b^	1.08 ± 0.01 ^c^	1.07 ± 0.03 ^c^
Cryptorchid	0.30 ± 0.01 ^b^	0.86 ± 0.10 ^b^	0.96 ± 0.01 ^b^	0.83 ± 0.02 ^b^
Contrascrotal	0.36 ± 0.01 ^a^	1.32 ± 0.14 ^a^	1.06 ± 0.01 ^a^	1.85 ± 0.05 ^a^

^a,b,c^ Means in same column with different superscripts differ (*p* < 0.001).

## Data Availability

The data that support the findings of this study are available on request from the corresponding author. The data are not publicly available due to privacy or ethical restrictions.
